# Sexual dimorphism and the impact of aging on ball rolling-associated locomotor behavior in *Drosophila*

**DOI:** 10.1242/bio.060609

**Published:** 2024-11-13

**Authors:** Gupta Soyam, Nisha N. Kannan

**Affiliations:** Chronobiology Laboratory, School of Biology, Indian Institute of Science Education and Research, Thiruvananthapuram, Kerala 695551, India

**Keywords:** Ball rolling, Sexual dimorphism, Locomotor activity, Aging, Behavior

## Abstract

Insects exhibit a remarkable ability to interact with inanimate objects to facilitate essential behaviors such as foraging, reproduction, shelter building, and defense. In this study, we assessed whether *Drosophila* interacted with inanimate objects when they were suspended on their wings and provided with a thermocol ball (foam ball). *Drosophila* indeed exhibited ball rolling behavior. We further examined the sexual dimorphism in this ball rolling-associated locomotor behavior. We carried out a ball rolling assay using 3-day-old male and female *w^1118^* flies and measured the duration for which the flies could roll the ball without dropping it within a 10 min period. The ball was returned to the flies whenever they dropped it, and we calculated the number of times the ball was dropped within the 10 min duration. Females exhibited a longer ball holding duration than males. We also observed a decrease in ball holding duration and an increase in the number of times the ball was dropped by 15-day-old male and female flies than their younger counterparts. These results suggest sexual dimorphism and age-dependent alterations in *Drosophila* ball rolling-associated locomotor behavior.

## Teaser

**Summary:** Fruit flies exhibited sexual dimorphism and age-related changes in ball rolling-associated locomotor behavior when they were suspended on their wings and provided with a thermocol ball (foam ball).

## INTRODUCTION

Insects rely on coordinated locomotion in a wide range of daily fundamental activities like foraging, mating behavior, escape responses, aggression and social interactions. Walking behavior in *Drosophila* combined with their ability to process various visual cues enables them to navigate towards salient objects in the environments including food, shelter and other essential resources (Robie et al., 2010). Male and female fruit flies share numerous similarities in locomotor behavior, differences may arise due to sex-specific roles related to courtship (Ferveur, 2010), mating (Maklakov and Bonduriansky, 2009), and olfactory signaling (Van Der Goes Van Naters and Carlson, 2007). Studies have shown that locomotor activity may differ between male and female fruit flies (Helfrich-Förster, 2000). In the afternoon, when sunlight and temperatures are high, flies reduce their locomotor activity and increase sleep (siesta) to avoid the energy expenditure caused by the heat. Sex differences in the amount of sleep that occurs during the day has been studied extensively. The amount of time that females spend sleeping is just 40% of that of the male flies (Huber et al., 2004). Sexual dimorphism in ‘siesta’ is probably because females need to be awake in order to forage for food to meet their reproductive nutritional requirements and choose locations for laying eggs, whereas males can spend more time saving energy and remain motionless as a strategy to evade physical and predatory threats (Isaac et al., 2010).

Studies on age-related changes in *Drosophila* suggest that age can impact various aspects of their behavior, including walking (Coen et al., 2014), olfaction (Tamura et al., 2003), and circadian rhythmicity (Grotewiel et al., 2005). Older flies may exhibit alterations in walking speed and less exploratory behavior compared to younger flies. Older flies may show altered feeding preferences and decreased food intake than younger flies (Kubrak et al., 2014). Aging can affect locomotor activity in fruit flies, including reduced flight capability (Privalova et al., 2021), decreased endurance, and impaired muscle coordination (Demontis et al., 2013; Miller et al., 2008) compared to younger flies.

Insects interact with and manipulate inanimate objects in diverse ways. For instance, social insects such as ants and termites manipulate materials like soil and leaves to construct nests and storage areas. Certain species of beetles roll balls of dung to lay eggs for females or to use as food sources (Holter, 2000). Ball rolling behavior requires simultaneous coordination of multiple muscles and limbs to perform walking, pushing the ball while maintaining balance (Khaldy et al., 2020). It also involves interplay of sensory processing, motor control, navigation, and learning mechanisms, all orchestrated by complex neuronal circuits (el Jundi et al., 2019). *Drosophila* typically does not exhibit ball rolling behavior like other insects such as cow dung beetles (Baird et al., 2012). However, fruit flies require a certain level of handling skills and inanimate object manipulation to extract nutrients from fruits, vegetables, and decaying organic materials. *Drosophila* consume decaying organic matter by landing on it and using their legs, which consist of taste neurons containing receptors for sugars, water, and bitter compounds (Meadows, 2009). Female fruit flies select suitable substrates for laying their eggs, often preferring decaying fruits or other organic matter (Yang et al., 2008). This behavior also involves the choice of a suitable substrate by interacting with inanimate objects.

In the current study, we used the fruit fly *Drosophila melanogaster* and assessed whether fruit flies interact with inanimate objects when they are suspended on their wings and provided with a thermocol ball (foam ball)*. Drosophila* have a small body size, which offers several advantages for the self-motion and movement control and also allows for the quantitative analysis of movement across the body in both a naturalistic as well as a lab environment (Chiappe, 2023). The results of our present study showed that fruit flies indeed exhibit ball rolling-associated locomotor behavior while interacting with an inanimate object like a thermocol ball (foam ball) and suggests a complex behavior that was perhaps not previously fully understood. Our results also showed that fruit flies exhibit sexual dimorphism in this ball rolling-associated locomotor behavior. Older flies exhibited a notable decrease in the duration that they could hold and rotate the ball, indicating potential alterations in this behavior with aging.

## RESULTS

### Sexual dimorphism and temporal variation in ball rolling-associated locomotor behavior

**Fig. 1.**
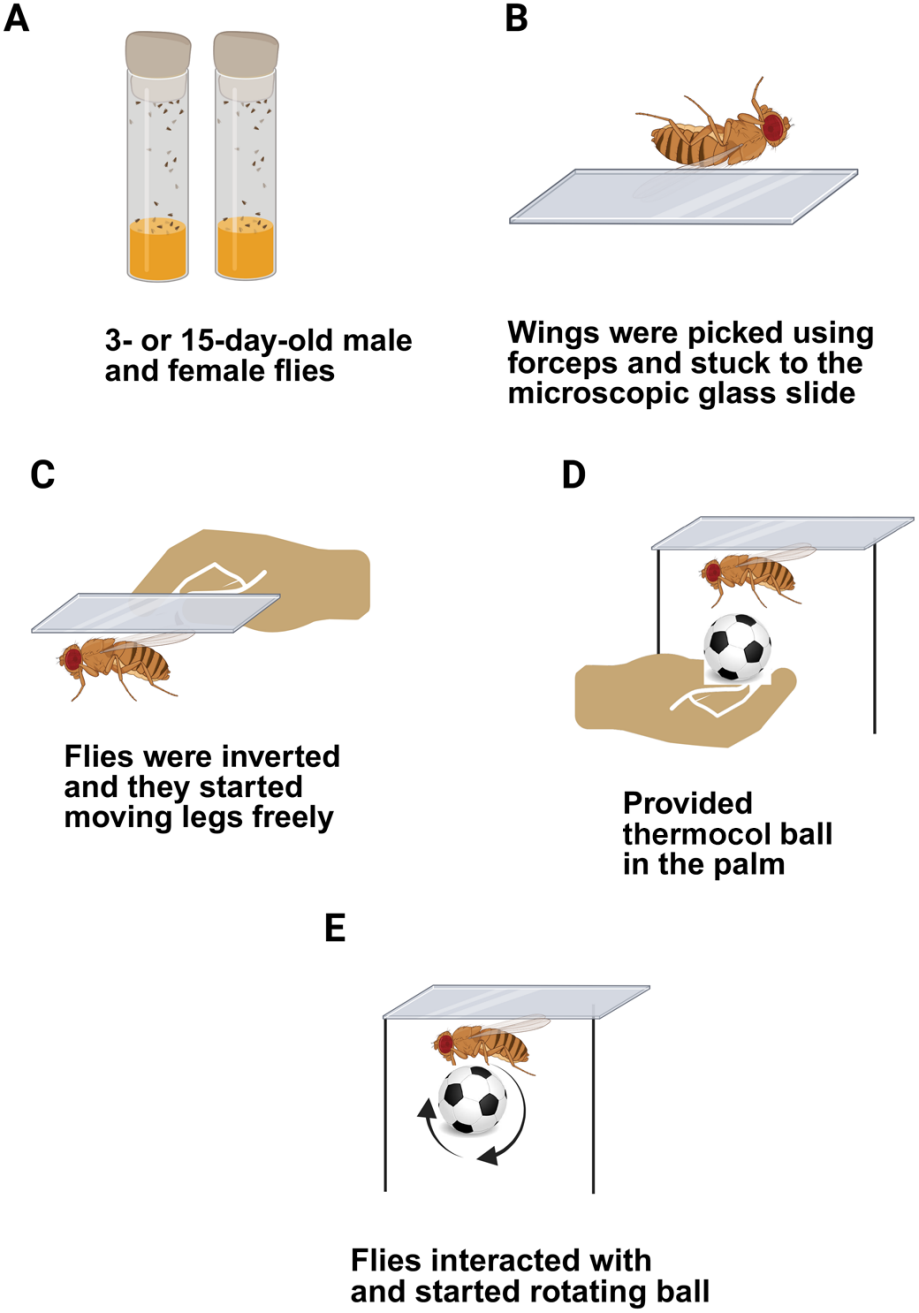
**Schematic representation of ball rolling assay.** (A) 3- or 15-day-old male and female *w^1118^* strains of *Drosophila* were anesthetized. (B) The wings were gently held with forceps, and by using double-sided foam tape the wings were attached to a microscope glass slide. (C) The slide was inverted to suspend the flies when they recovered from anesthesia and had regained mobility by moving their legs freely. (D) A clean thermocol ball (3 mg) was placed in the palm of the hand and brought closer to the flies so that they held and interacted with it. (E) Ball rolling activity of flies was recorded for a duration of 10 min to quantify two variables.

**Fig. 2.**
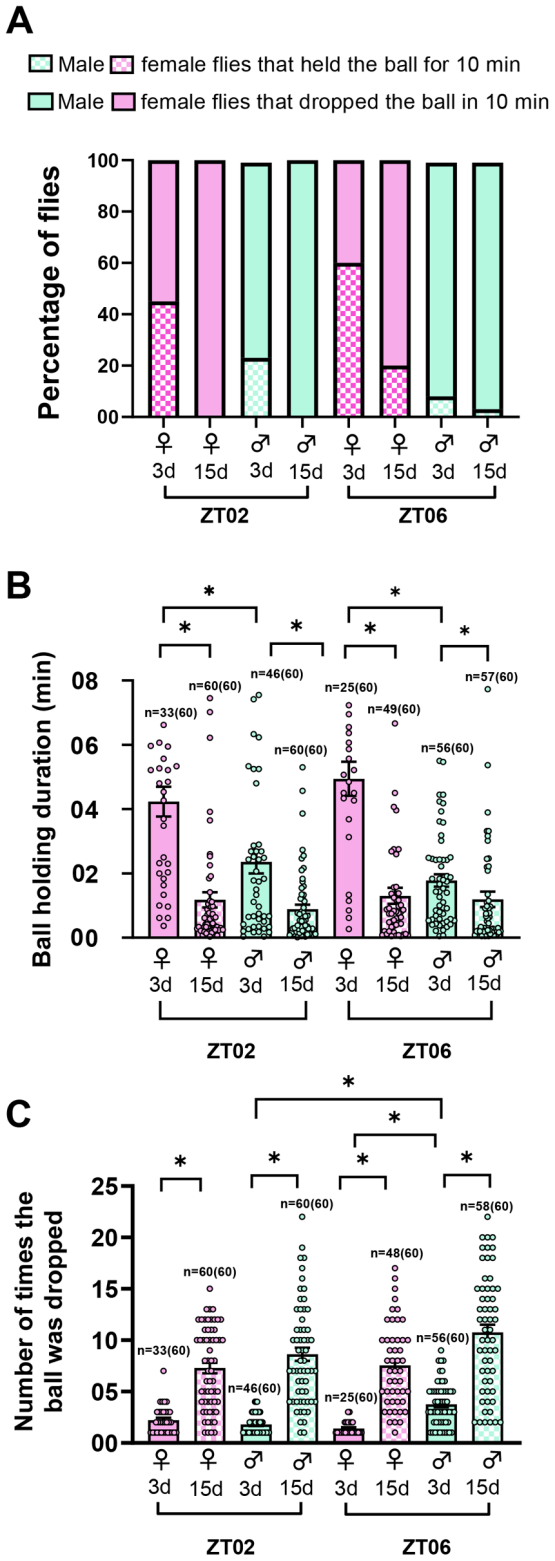
**Sexual dimorphism and effect of age in ball rolling-associated locomotor behavior.** (A**)** Percentage of 3-day-old or 15-day-old w*^1118^* male and female flies that were able to hold the ball for 10 min and flies that dropped the ball in 10 min at ZT02 and ZT06. (B) Average ball holding duration in 10 min by 3-day-old and 15-day-old w*^1118^* females and males at ZT02 and ZT06. (C) The average number of times the ball was dropped in 10 min by 3-day-old and 15-day-old *w^1118^* females and males at ZT02 and ZT06. The time points at which males exhibit significant differences with respect to females are indicated with asterisks.

To assess sexual dimorphism and temporal variation in ball rolling-associated locomotor behavior, we carried out a ball rolling assay using 3-day-old *w^1118^* male and female flies at ZT02 and ZT06 (the methodology is shown in Fig. 1). We estimated how long the flies were able to hold and roll the ball without dropping it and further estimated the average number of times the ball was dropped in 10 min during the ball rolling session. Among the 60 flies examined at ZT02, 45% female flies (27 of 60) were able to hold the ball for 10 min without dropping it, whereas only 23% of male flies (14 of 60) could do so. At ZT06, 60% females (36 of 60) were able to hold the ball for 10 min. In contrast, only 8% of males (5 of 60) achieved the same feat (Fig. 2A). To assess the effect of age on temporal variation and sexual dimorphism in ball rolling behavior, we carried out a ball rolling assay at ZT02 and ZT06 by using 15-day-old *w^1118^* male and female flies and compared it with that of 3-day-old flies. While 46% of 3-day-old female flies (28 of 60) at ZT02 were able to hold the ball for 10 min, none of the 15-day-old females (0 of 60) could do so. Among 3-day-old males, 23% of flies (14 of 60) managed to hold the ball for 10 min at ZT02, whereas none of the 15-day-old males (0 of 60) were able to hold the ball for 10 min. At ZT06, 60% of 3-day-old females (36 of 60) were able to hold the ball for 10 min, whereas only 20% of 15-day-old females (12 of 60) could do the same. For 3-day-old males, 8% (5 of 60) of flies managed to hold the ball for 10 min at ZT06 in contrast to just 3% of 15-day-old males (2 of 60) (Fig. 2A). When comparing 15-day-old males and females at ZT02 and ZT06 none of them at ZT02 were able to hold the ball for 10 min without dropping it, whereas a few of them were able to do so at ZT06.

We calculated the AIC value using logistic regression analysis, considering sex, time (ZT), and age as independent factors for this experiment. The lowest AIC value was observed for the sex×time×age interaction, indicating that the interplay between these factors affects the flies' ability to hold the ball for 10 min. In this interaction, the *P*-values for sex and age were significant, while time was not (Table 1). However, *P*-value for time was significant for sex×time interaction. In addition, when these three factors were analyzed individually, significant *P*-values were obtained for all three (Table 1). Taken together, these results suggest that while the sex, time and age influence the flies' ability to hold the ball, time may play a lesser role.

**Table 1.**
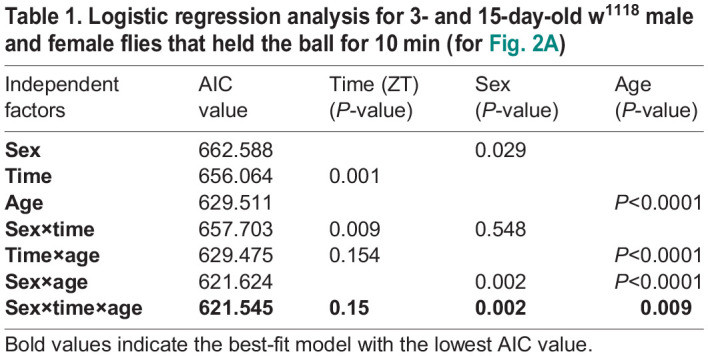
Logistic regression analysis for 3- and 15-day-old w^1118^ male and female flies that held the ball for 10 min (for Fig. 2A)

### Effect of sex and age on ball holding duration

We further examined the 60 flies that dropped the ball in the 10 min session and noted the time at which these flies dropped the ball for the first time within the 10 min and defined this duration as the ball-holding duration. 3-day-old male flies did not exhibit any significant difference in the ball-holding duration between ZT02 and ZT06. Whereas at both the time points ZT02 and ZT06, 3-day-old females held the ball for a longer duration than males (Fig. 2B, Kruskal–Wallis test followed by Dunn's multiple comparisons test, *P*<0.05 for ZT02 and ZT06). While considering 15-day-old flies, both male and female flies exhibited a significant decrease in ball-holding duration at both ZT02 and ZT06 compared to 3-day-old flies (Fig. 2B, Kruskal–Wallis test followed by Dunn's multiple comparisons test, *P*<0.001 for females at ZT02 and ZT06, *P*<0.05 for males at ZT02 and ZT06). In 3-day-old flies, females held the ball for a longer duration than males at both ZT02 and ZT06, indicating sexual dimorphism in ball-holding duration, which was lost in older flies. We calculated the AIC value using linear regression analysis, with sex, time (ZT), and age as independent factors. The lowest AIC value was observed for the sex×age interaction not for sex×time×age indicating that the interplay between sex and age affects ball holding duration (Table 2). When these three factors were analyzed individually, significant *P*-values were obtained for sex and age, but not for time, suggesting that time may not influence ball holding duration, whereas the sex and age do (Table 2).

**Table 2.**
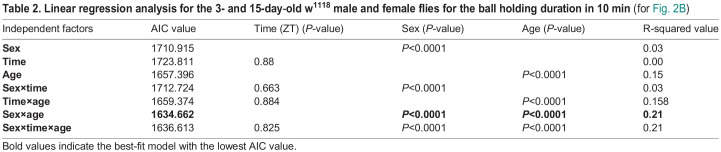
**Linear regression analysis for the 3- and 15-day**-**old w^1118^ male and female flies for the ball holding duration in 10 min (for Fig. 2B)**

After the flies initially dropped the ball, it was consistently returned to them each subsequent time they dropped it. We then recorded the total number of times the ball was dropped in 10 min to indirectly infer whether their muscle strength and ability to control movements varied between males and females, as well as how these abilities change with age. When we compared 3-day-old males and females at ZT02, there was no difference observed in the average number of times the ball was dropped. We also compared 3-day-old males and females at ZT06 and it was observed that the males dropped the ball more times than females (Fig. 2C, Kruskal–Wallis test followed by Dunn's multiple comparisons test, *P*<0.0001). 15-day-old male and female flies exhibited a significant increase in the number of times the ball was dropped at both ZT02 and ZT06 than 3-day-old flies (Fig. 2C, Kruskal–Wallis test followed by Dunn's multiple comparisons test, *P*<0.001 for males at ZT02 and *P*<0.001 for females at ZT02, *P*<0.001 for males at ZT06 and *P*<0.001 for females at ZT06). When comparing 3-day-old males and females at ZT06, it was observed that males dropped the balls more frequently than females. However, this sexual dimorphism in ball rolling-associated locomotor behavior was no longer present in older flies. The AIC value was calculated using logistic regression analysis, considering sex, time (ZT), and age as independent factors. The lowest AIC value was observed for age, indicating that this factor significantly affects the number of times the ball was dropped within 10 min (Table 3).

**Table 3.**
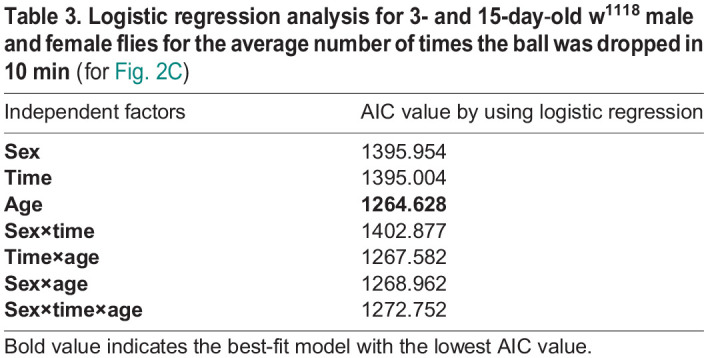
**Logistic regression analysis for 3- and 15-day**-**old w^1118^ male and female flies for the average number of times the ball was dropped in 10 min (for Fig. 2C)**

### Sexual dimorphism and age-dependent changes in locomotor activity levels in *Drosophila*

**Fig. 3.**
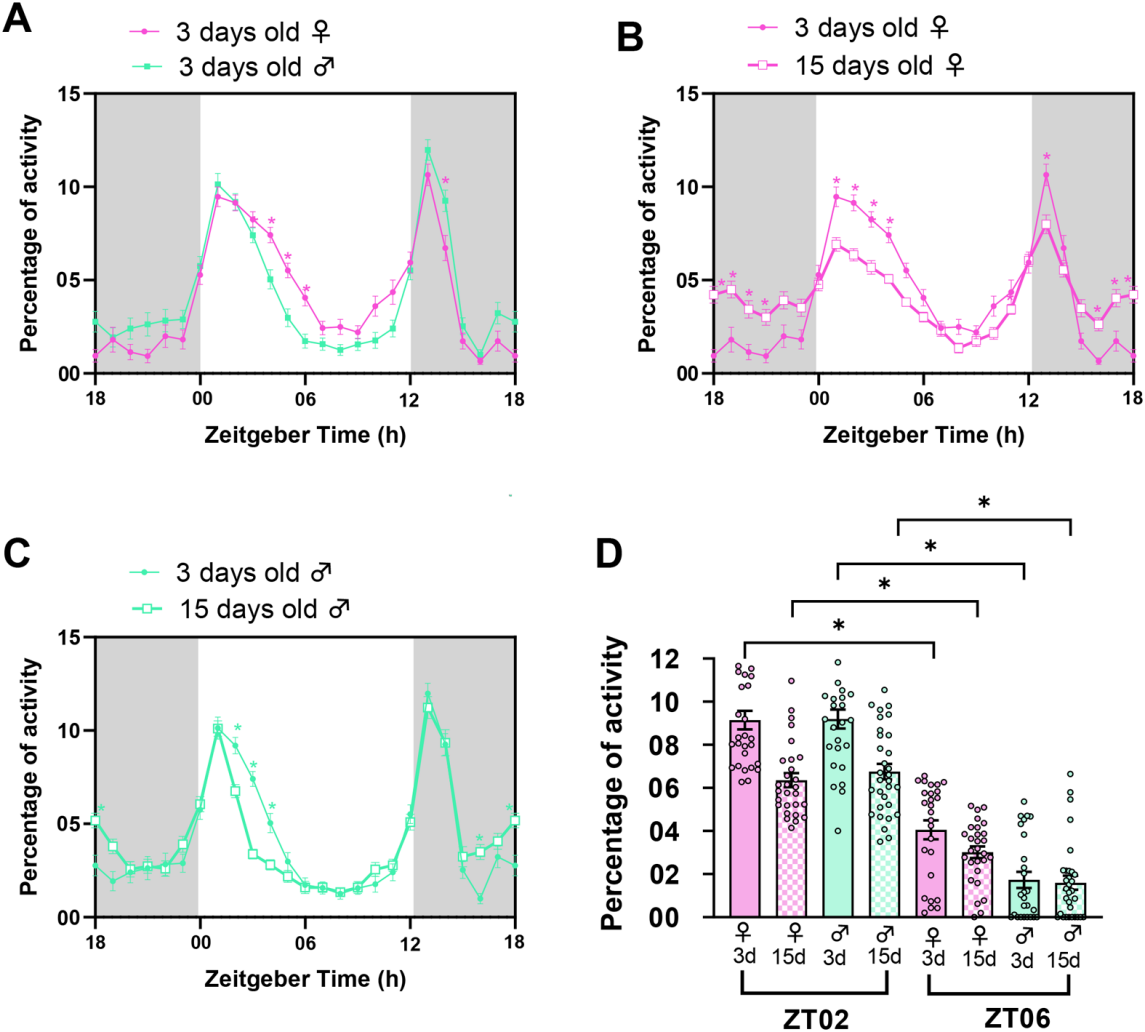
**Effect of sex and aging on locomotor behavior.** (A) Percentage of activity of 3-day-old w*^1118^* male and female flies are plotted against ZT. Grey shaded region indicates the dark phase under LD. (B,C) Percentage activity of 3-day-old and 15-day-old w*^1118^* male and female flies are plotted against ZT. (D) Percentage activity at ZT02 and ZT06 by 3-day-old and 15-day-old females and males.

The sexual dimorphism observed in ball rolling-associated locomotor behavior could be possibly due to the variations in their locomotor activity levels. Hence, we recorded the locomotor activity of 3-day-old *w^1118^* male and female flies with a *Drosophila* activity monitor (DAM) to examine the sexual dimorphism and temporal variation in locomotor activity. It was found that 3-day-old male flies exhibit a significant decrease in locomotor activity at ZT04–06 than females (Fig. 3A, two-way ANOVA test followed by Šídák's multiple comparisons test, *P*<0.05 for all the time points). In addition, both males and females exhibited a significant decrease in locomotor activity at ZT06 than at ZT02 (Fig. 3D, Kruskal–Wallis test followed by Dunn's multiple comparisons test, *P*<0.0001 for males and, *P*<0.001 for females). These results suggest that *Drosophila* exhibit sexual dimorphism and diurnal variation in their locomotor activity.

To assess whether age-dependent changes in locomotor activity may affect the ball rolling-associated locomotor behavior, we recorded the locomotor activity of 15-days-old *w^1118^* male and female flies. Both 15-day-old males and females exhibited an increase in the activity during certain time points in the dark phase and a decrease in the activity during some parts of the light phase when compared to 3-day-old flies (Fig. 3B,C; ANOVA followed by Šídák's multiple comparisons test, *P*<0.05 for all the time points). In addition, both 15-day-old males and females exhibited significant decrease in locomotor activity at ZT02 compared to 3-day-old flies (Fig. 3B,C, ANOVA test followed by Šídák's multiple comparisons test, *P*<0.05 for both males and females). However, no significant difference in locomotor activity was found between 3- and 15-day-old males and females at ZT06 (Fig. 3B,C). Additionally, both 15-day-old males and females exhibited a significant decrease in locomotor activity at ZT06 compared to ZT02 (Fig. 3D, Kruskal–Wallis test followed by Dunn's multiple comparisons test, *P*<0.05 in 15-day-old females at ZT02 versus ZT06, *P*<0.001 in 15-day-old males at ZT02 versus ZT06), indicating the temporal variation in locomotor activity persists in older flies. We calculated the AIC value using linear regression analysis with sex, time (ZT02 and 06), and age as independent factors. The lowest AIC value was observed for the sex×time×age interaction, indicating that the interplay between these factors affects the changes observed in the locomotor activity at ZT02 and ZT06 (Table 4).

**Table 4.**
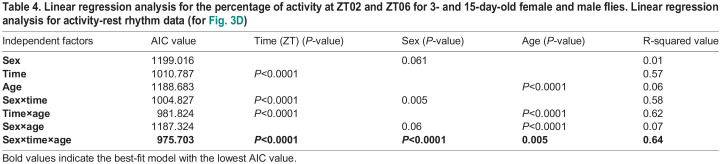
Linear regression analysis for the percentage of activity at ZT02 and ZT06 for 3- and 15-day-old female and male flies. Linear regression analysis for activity-rest rhythm data (for Fig. 3D)

## DISCUSSION

In fruit flies, walking and climbing are the crucial behaviors for exploring their environment, finding food, mating, and laying eggs. Fruit flies primarily move by walking on solid surfaces. They use their six legs to navigate and explore their environment (Kain et al., 2013). Climbing ability makes fruit flies highly versatile in navigating complex environments (Currier and Nagel, 2020). This is vital for accessing food, such as overripe fruits, which often hang in places that require climbing. The ability to switch between walking, climbing, and flying provides them with versatile strategies to deal with various inanimate obstacles and surfaces. The locomotion of *Drosophila* involves complex interactions with inanimate objects in their environment. In the present we showed that *Drosophila* interact with inanimate objects and exhibit sexual dimorphism in ball rolling-associated locomotor behavior when they were provided with a thermocol ball.

This unique and complex ball rolling-associated locomotor behavior indeed involves simultaneous leg coordination and motor control to keep walking on uneven surfaces, pushing the ball to a suitable position to roll, and prevent the ball from toppling over. The flies used in this experiment were not climbing or balancing, because they were attached to microscope slides. However, our study could still be relevant to the climbing behavior of wild-type flies. Sexual dimorphism in *Drosophila*, particularly regarding walking and climbing ability, refers to differences in physical performance between male and female fruit flies (Millington and Rideout, 2018). Males and females have distinct walking patterns. Males exhibit more dynamic climbing ability and variable walking speed and pattern during chasing and courtship (Billeter et al., 2006; Pan et al., 2011). Males are typically smaller than females (Millington and Rideout, 2018), differences in leg morphology and muscle structure might also influence their walking dynamics. While males are often noted for their dynamic and variable walking pattern and climbing ability associated with courtship and aggression (Thiem et al., 2024), females also exhibit increased locomotor activity, particularly for foraging and oviposition (Aranha and Vasconcelos, 2018). Females utilize their climbing ability to explore and assess the egg-laying sites and thus ensure the safety and suitability of these locations for their offspring (Yang et al., 2008). These differences in walking and climbing behavior between sexes could potentially play a role in the sexual dimorphism observed in *Drosophila* ball rolling-associated locomotor behavior.

*Drosophila* exhibit a bimodal pattern of activity with the morning and evening peaks and a significant drop in the activity during the midday, known as the siesta (Chiu et al., 2010). This activity pattern is influenced by the light-dark cycle as well as by the circadian clock. Studies showed notable differences between male and female *Drosophila* in their bimodal activity pattern where males exhibit enhanced sleep during the midday compared to females likely due to differences in their hormones and energy demands (Helfrich-Förster, 2000). Possibly, this time-dependent decrease in activity levels in male flies may be attributed to sexual dimorphism observed in the ball rolling-associated locomotor behaviour.

The results of our study suggest that with increasing age, the ball rolling ability diminishes in *Drosophila*. While young flies display well-defined activity-rest rhythm, aging flies may experience disruptions in this with increased fragmentation of activity bouts, alter the phase of morning and evening activity peaks and reduced activity levels (Koh et al., 2006). These changes may reflect alterations in the underlying circadian clock and the sleep neural circuit (Kondratova and Kondratov, 2012). Additionally, reduction in walking speed, distance covered, and decline in exploratory behavior associated with age may also be linked to these changes in the ball rolling behavior. Typically, young flies exhibit robust climbing behavior. However, as flies age, their climbing performance tends to deteriorate, with older flies showing reduced climbing speed and decreased climbing success rates compared to younger flies (Thiem et al., 2024). Age-related impairments in motor function may contribute to decreased climbing ability observed in older flies. Synaptic deterioration, neuronal cell death, and changes in neurotransmitter signaling, can impair motor coordination and climbing ability in older flies (Azpurua et al., 2018). Therefore, it is probable that age-related decrease in muscle strength, changes in neuromuscular coordination, and declining climbing ability and locomotor activity changes may also play a role in the potential alterations seen in the ball rolling-associated locomotor behavior of older flies.

Our study elucidated the sexual dimorphism and age dependent alterations in ball rolling-associated locomotor behavior in *Drosophila*. But this ball rolling behavior is not frequently observed in wild *Drosophila*, as it is in the case of cow dung beetles. This behavior resulted from the tethered fly being given a ball. Therefore, we characterized it as ball rolling-associated locomotor behavior rather than actual ball rolling observed in other insects. Walking behavior in *Drosophila* is studied extensively by employing sophisticated techniques (Chiappe, 2023; Creamer et al., 2018; Feng et al., 2020; Tsai and Chou, 2019; Agrawal et al., 2020; Gargano et al., 2005). Our study differs from those existing walking behavior studies on *Drosophila* and other insects. Because the ball rolling-associated locomotor behavior we studied involves coordinating legs simultaneously and controlling motor functions to walk on uneven surfaces, climb, and maintain balance while positioning and rolling the ball without dropping it, it is indeed unique and complex. Further, exploring the coordination between the brain, nervous system, and motor functions in executing locomotor behaviors associated with ball rolling may provide novel insights into the brain function and neural circuits that drive behavior.

## MATERIALS AND METHODS

### Fly line and maintenance

This study used *w^1118^* (BDSC #5905) flies for all the experiments. Fly stocks were maintained on a standard cornmeal medium in a cooled incubator (Percival Scientific) under 12 h (h) light:12 h dark cycle (LD) at 25°C temperature, ∼450 lux light intensity and 70±5% humidity. The lights in the incubator came on at Zeitgeber Time 00 (ZT 00) and went off at ZT 12. Fifty 1st instar larvae were collected in fresh food vials, to avoid overcrowding, within 2–3 h of hatching. Freshly emerged male and female flies were collected (15–20 per vial), and 3-day-old adult flies were used for the experiments to assess sexual dimorphism and temporal variation in ball rolling-associated locomotor behavior. 15-day-old adult flies were also used to check the effect of aging on ball rolling-associated locomotor behavior.

### Ball rolling assay

3- or 15-day-old flies were anesthetized with carbon dioxide (CO_2_) and positioned under a microscope. The wings of the anesthetized flies were held gently with forceps (Alis precision diamond tweezer) and by using double-sided foam tape (such as Magnus Deal double sided foam tape), the wings were attached to a microscope glass slide (Borosil-9100D01). We ensured that only the wings were attached to the slide and that the rest of the fly's body remained free. Immobilizing the wings while leaving the rest of the body free allowed natural movement in the flies. As soon as the flies recovered from anesthesia and regained mobility by moving their legs freely, the slide was inverted to suspend the flies. A clean thermocol ball (foam ball), made from polystyrene, a lightweight and porous material commonly known as styrene foam (weight, 3 mg) was placed on the palm of the researcher and brought closer to the flies so that they could interact with it and hold it (Fig. 1; Movie 1). During the experiment, each fly was provided with a new ball after every experiment to prevent contamination or potential bias in the results due to leftover olfactory or tactile cues. Upon holding the ball, the behavior of the flies was observed and the video was recorded using a 50 MP AI camera.

This recorded video was used to quantify the ball rolling-associated locomotor behavior of flies as the following three variables. (i) Percentage of flies that held the ball without dropping for 10 min. (ii) We further analyzed the flies that dropped the ball in the 10 min session and examined the time at which these flies dropped the ball for the first time. This was quantified as the ball holding duration. (iii) Each time the flies dropped the ball, it was provided to them again and the number of times the ball was dropped within the 10 min duration of ball rolling was recorded. We did this assay by using male and female *w^1118^* strains of *Drosophila* to assess the sexual dimorphism in ball rolling-associated locomotor behavior. We did the assay at time point ZT02 (2 h after lights-on) and ZT06 (mid-day) to assess the temporal variation in this behavior. In this assay, 60 flies per treatment group were used, which included 60 females and 60 males from each age group (3 days and 15 days), resulting in a total of 240 flies for the experiments at ZT02. As for the time-of-day assay, we used different sets of flies for each time point (ZT02 and ZT06). This means that a new group of 240 flies (120 females and 120 males) was tested at ZT06, bringing the total number of flies used in the time-of-day assay to 480. We did not use the same individual flies at multiple time points. Because we used different flies at each time point, the data from ZT02 and ZT06 are independent. After the experiment, flies were discarded in the designated fly morgues where 70% ethanol was used for disposal.

### Locomotor activity assay

The *Drosophila* Activity Monitor (Model DAM2 for 5 mm tube) system (Trikinetics, Waltham, MA, USA) was used to record the locomotor activity of *Drosophila*. Individual flies were placed within a 5 mm glass tube, and their activity was measured by means of infrared beam breaks over the course of 24 h. 3- and 15-day-old flies were used and their activity was recorded under LD at 25°C in a cooled incubator (Panasonic MIR-154) with a 1 min bin interval. 28–32 flies were used for the locomotor activity recording under each condition. The light intensity was maintained at ∼400 lux. The activity count for each individual fly was recorded for 24 h, and activity at every 1 h was normalized to the total activity of the day, multiplied by 100 to obtain the percentage of activity. Separate flies were used for both the locomotor activity recording and the ball rolling assay.

### Statistical analysis

The number of times the ball was dropped, the duration for which the flies held the ball without dropping and the activity level at ZT02 and ZT06 were analyzed for normal distribution by using Shapiro-Wilk test. Normally distributed data were analyzed by two-way ANOVA test followed by Šídák's multiple comparisons test. Kruskal–Wallis test followed by Dunn's multiple comparisons test was used for data that were not normally distributed. Results were regarded as statistically significant if the *P*-value was <0.05. To determine whether sex, time of day, or age affected the percentage of flies that could hold the ball for 10 min, we performed a logistic regression analysis. We also applied the Akaike Information Criterion (AIC, Akaike, 1969) to select the best-fit model with the lowest AIC value and identify significant independent factors. Similarly, to evaluate whether sex, time of day, or age influenced the ball-holding duration and locomotor activity, we performed a linear regression analysis, we used AIC to select the best-fit model and determine which factors were significant. To evaluate whether sex, time of day, or age affected the number of times flies dropped the ball, we used logistic regression analysis followed by AIC calculation to determine the best model and significant factors. Logistic regression analysis has been done for the data whose output is discrete and linear regression has been done for continuous output data. The graphs were plotted using GraphPad Prism8 software. The graphs were plotted with mean and error bars in all the graphs representing the standard error of the mean (s.e.m.).

## Supplementary Material

10.1242/biolopen.060609_sup1 Supplementary information
