# Supplementary material for: Sexual dimorphism and the impact of aging on ball rolling-associated locomotor behavior in Drosophila
Source: Biol Open. 2024 Nov 13;13(11):bio060609. doi: 10.1242/bio.060609 (PMC11583920; doi:10.1242/bio.060609)
Supplement: Supplementary information [file biolopen-13-060609-s1.pdf]

Dataset 1. Raw data

Available for download at
https://journals.biologists.com/bio/article-lookup/doi/10.1242/bio.060609#supplementary-data

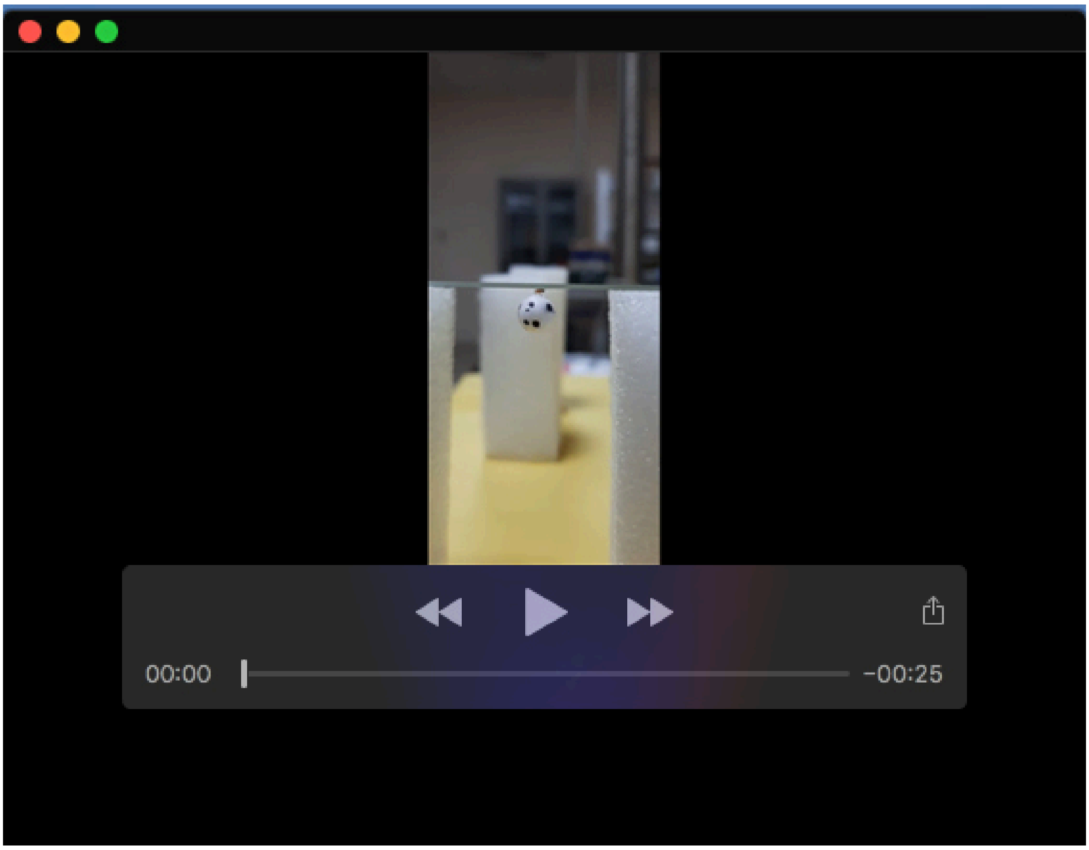

Movie 1.
